# A Machine-Learning-Based Approach to Informing Student Admission Decisions

**DOI:** 10.3390/bs15030330

**Published:** 2025-03-07

**Authors:** Tuo Liu, Cosima Schenk, Stephan Braun, Andreas Frey

**Affiliations:** Institute of Psychology, Goethe University Frankfurt, 60323 Frankfurt am Main, Germany; schenk@psych.uni-frankfurt.de (C.S.); braun@psych.uni-frankfurt.de (S.B.); frey@psych.uni-frankfurt.de (A.F.)

**Keywords:** enrollment management, admissions, enrollment yield, machine learning, predictive modeling, statistical uncertainty

## Abstract

University resources are limited, and strategic admission management is required in certain fields that have high application volumes but limited available study places. Student admission processes need to select an appropriate number of applicants to ensure the optimal enrollment while avoiding over- or underenrollment. The traditional approach often relies on the enrollment yields from previous years, assuming fixed admission probabilities for all applicants and ignoring statistical uncertainty, which can lead to suboptimal decisions. In this study, we propose a novel machine-learning-based approach to improving student admission decisions. Trained on historical application data, this approach predicts the number of enrolled applicants conditionally based on the number of admitted applicants, incorporates the statistical uncertainty of these predictions, and derives the probability of the number of enrolled applicants being larger or smaller than the available study places. The application of this approach is illustrated using empirical application data from a German university. In this illustration, first, several machine learning models were trained and compared. The best model was selected. This was then applied to applicant data for the next year to estimate the individual enrollment probabilities, which were aggregated to predict the number of applicants enrolled and the probability of this number being larger or smaller than the available study places. When this approach was compared with the traditional approach using fixed enrollment yields, the results showed that the proposed approach enables data-driven adjustments to the number of admitted applicants, ensuring controlled risk of over- and underenrollment.

## 1. Introduction

Every year, tens of millions of people around the world apply to study at universities. Subject areas such as medicine, psychology, and economics are in especially high demand (National Center for Education Statistics, n.d.). When processing these applications, one recurring task of universities is to decide whether applicants to a designated study program are admitted or not. The ratio of the number of applicants admitted *A* to the total number *T* of applicants to a study program in one selection period is called the admission rate *AR*, which can be represented as a percentage as follows:

(1) $$AR=\frac{A}{T}\times 100$$

The lower the *AR*, the more selective the access to the respective study program. Study programs such as medicine, law, engineering, and psychology at highly prestigious universities like Ivy League schools in the United States (US) often have an *AR* of 10% or below (Nietzel, 2022).

In practice, different sources of information are used to make admission decisions. Universities in the US and several other countries typically use a holistic approach that integrates different sources of information such as the grade point average (GPA), standardized test scores (e.g., SAT, GRE), essay grades (evaluations of written personal statements), recommendation letters (assessments from teachers or mentors), and interviews (personal evaluations of applicants’ suitability and motivation; see more in Briihl & Wasieleski, 2004). Universities in other countries, such as Germany, rely more on numerical information such as the numerus clausus or domain-specific tests (Unangst, 2019). In rare cases, all applicants who satisfy one or more selection criteria are admitted. Even though such a criterion-referenced decision may be desirable, it is seldom used because such definite criteria are hard to define. Furthermore, the procedure means that the number of applicants who finally enroll (denoted as *E* for the rest of this paper) cannot be determined before they complete their enrollment. This is infeasible for most study programs because the number of study places is typically fixed, which means that the number of students who enroll needs to be effectively controlled. Therefore, the predominant case is the admission of a fixed number of applicants who meet the selection criteria best.

To accomplish such a selection, the applicants are typically rank-ordered with regard to a single criterion or combined selection criteria. If all applicants accepted the study place they were offered, the process would be simple: Starting with Rank 1, as many applicants would be admitted as there were study places (denoted as *SP* for the rest of this paper) available. But in practice, a relatively large number of admitted applicants do not enroll in the study program, so that A<SP. There are various potential reasons for this: An individual may have applied to several different universities and received several admissions, of which only one can lead to actual enrollment. Other possible reasons are the decision to study another subject area, being called for military service, taking up a job or starting with vocational training, an illness or that of a close relative, the birth of a child, or the decision to take a longer vacation before studying. Because reasons like these contribute to the fact that universities typically often enroll less than 50% of the applicants they admit, the enrollment yield *EY* needs to be considered in the admission process, which is defined as follows:

(2) $$EY=\frac{E}{A}\times 100$$

The *EY* is the proportion of applicants admitted to the applicants who actually enroll. Rearranging this formula into

(3) $$A=\frac{E}{EY}\times 100$$

seems to be a simple solution for determining *A* when the number of study places is fixed so that E=SP and when a good estimate for *EY* is available. If, for example, a study program has a fixed number of 120 study places to fill and last year’s enrollment yield was 40% and can be expected to be the same this year, then accepting 120/40×100=300 applicants seems straightforward. But this simple solution has four shortcomings. First, it is based on knowledge about the EY that may fluctuate between years. Second, it assumes that the probability of applicants enrolling after admission is invariant across applicants. There is considerable empirical evidence that this assumption is problematic (e.g., Fu et al., 2022; Lotfi & Maki, 2018). Third, it only provides a point estimate for *A* and does not provide any information about its statistical uncertainty. Fourth, and related to the previous point, there is no information on the probability P(E>SP|A) that more applicants will enroll than there are study places available when *A* applicants are admitted (and the complement (P[E≤SP∣A]) that a maximum of as many applicants enroll as there are study places available conditional upon *A*).

A lack of knowledge of these probabilities, combined with the fact that it is problematic or even impossible for universities to enroll more applicants than there are study places available, means that the people responsible for deciding upon *A* are likely to be too cautious and to use an unnecessarily small value for *A*. This, in turn, will likely result in many open study places, making one or more additional admission rounds necessary. Even though such multiple admission rounds seem to be more a rule than an exception, they have two clear disadvantages. First, the applicants have a longer period of uncertainty, have less time to prepare for their studies (find housing, etc.), and sometimes even receive their acceptance letter so late that the first study term has already begun. This makes it harder for them to start their studies and is likely to extend the total time from enrollment to graduation, thereby increasing the average duration of study for students (Smith et al., 2002). Second, students not admitted in the first round but who have a low rank in the selection criteria will likely be offered a study place at another university. Therefore, the probability of obtaining the best students according to the selection criterion can be expected to be lower if they are admitted in subsequent admission rounds. In this paper, we suggest and examine a machine-learning-based approach to informing the decision about the number of students admitted to a study program. This approach has three objectives:

1. To predict the number of enrolled applicants *E* conditional on the number of admitted applicants *A*;
2. To derive the probability of too many applicants enrolling conditional on *A* and its complement;
3. To quantify the statistical uncertainty of the predicted number of enrolled applicants.

### The Suggested Approach

The suggested approach helps determine the number of admitted applicants in such a way that the enrollment of high-potential applicants (applicants with a low rank) is maximized while only allowing for a known probability of enrolling more applicants than there are study places. The approach has three steps. In Step 1, several machine learning models are trained on application data for the study program of interest from one or several preceding years to predict the individual probabilities that applicants will enroll when they are admitted to a study program. Previous studies have consistently highlighted the effectiveness of machine learning predictive models when applied to historical enrollment data (e.g., Bruggink & Gambhir, 1996; Goenner & Pauls, 2006; Shao et al., 2022; Shrestha et al., 2016; Walczak & Sincich, 1999; for a broader overview, see Li et al., 2023). Afterward, one of the estimated models from Step 1 is selected and used in Step 2. There, it is applied to the data from a cohort of current applicants for the same study program. The result is an array of the individual probabilities of the applicants actually enrolling if they are admitted. The individual probabilities are aggregated to derive an estimate of the number of enrolled applicants *E*. In Step 3, the statistical uncertainty of *E* and the probability of enrolling too many applicants P(E>SP|A) and its complement are determined for different values of *A*.

To be directly applicable in practical settings, three aspects need to be considered. First, only data available during a standard application process should be used because the collection of additional data would mean an additional burden and would make the application procedure more complicated. Second, the approach has to be in line with common data protection regulations. In this study, we used the comparatively strict General Data Protection Regulation (GDPR) of the European Union as the legal framework (European Parliament & Council of the European Union, 2016). Third, the statistical uncertainty of *E* for different values of *A*, P(E>SP|A) and its complement should be easily accessible for personnel without statistical training. For this purpose, an app was developed that could be run in a browser. This means that it can be used directly by the people typically assigned with the task of preparing the material for the admission decisions, such as personnel working in examination offices or admission officers.

A few studies have already suggested the use of predictive models to optimize student selection procedures (Goenner & Pauls, 2006; Lotfi & Maki, 2018; Shrestha et al., 2016; Walczak & Sincich, 1999). However, the approach we are proposing goes an important step further than the existing literature by quantifying the statistical uncertainty of *E* conditional on *A* and the predicted probabilities of enrolling and calculating the probability of admitting too many students and its complement.

The remainder of this paper is organized as follows. In the Section 2, the data used to exemplify the suggested approach, the three-step approach itself, the evaluation criteria, and the app are described. In the Section 3, first, different predictive models are compared with regard to the area under the curve (AUC) and the Brier score after calibration for the example data. The AUC is used to assess the discriminatory power of the prediction, while the Brier score evaluates the accuracy of the predicted probabilities. The model with the optimal balance between its discriminatory power and probability prediction accuracy is selected for further analysis. Then, a comparison of the predicted numbers of enrolled applicants with the empirically observed numbers is presented. The use of the app is visualized with screenshots. This paper ends with a summary of the main results and the conclusions.

## 2. Method

### 2.1. Data

In this paper, we exemplify the suggested approach for the typical admission process of a German university. This process has two distinct phases. In the first phase, staff from the examination office and the admission officers prepare the foundation for the decision-making. They compile a rank-ordered list of applicants based on one or several criteria, along with key metrics such as the total number of applications received *T*, the previous enrollment yield EY, and, if applicable, other relevant indicators. In the second phase, the final admission decisions are made by the people or committee in charge, using the rank-ordered list of the data from the first phase. To ensure compliance with data protection standards, all meetings in which the sensitive personal data of the applicants are discussed take place in confidential sessions, and the participants of these meetings are obliged to maintain discretion regarding these data. This structured two-phase procedure is more or less similar to that for most degree programs in Germany.

To demonstrate our approach, we used an authentic historical admission dataset from a Master in Psychology study program. Personal information was removed from this dataset or transformed in such a way that it was impossible to identify individual students. The analyzed dataset entails the details of all applicants, admitted applicants, and enrolled applicants over four academic years, from 2017 to 2020. Each year, around 1500 individuals apply for 1 of 120 study places. The committee in charge originally followed the traditional approach and selected 220 to 274 applicants per year in the first round and invited them to enroll in the study program. After the first round, the number of enrolled applicants typically was smaller than the number of study places, so another admission round was carried out. This was repeated up to a maximum of three admission rounds. The total number of applicants, the number of applicants admitted in the first round, the admission rate, the number of enrolled applicants, and the enrollment yield for the four years are shown in Table 1. The values shown in the table fluctuate considerably across the four years. The enrollment yield, for example, varies between 36.6% and 53.6%. This variation clearly indicates the necessity of a prediction model that takes individual enrollment probabilities into account. If the *EY* had been more or less constant across the four years, it would have been possible to apply Equation (3) to determine *A*.

The data from 2017 to 2019 were used to train the machine learning (ML) model, and the data from 2020 were used as the test dataset. Because the model served the purpose of predicting the individual probabilities of actually enrolling after admission, only the data for the applicants who were admitted were used for the training. For all of these individuals, information was available about whether they finally enrolled in the study program or not. Because our approach strove to fill the available study places in the first round as precisely as possible, the empirical data for the first round were used.

In order to enable readers to apply the proposed approach themselves, we made the R code and a demo dataset available in the OSF repository https://osf.io/qsvcj/?view_only=9b27e0c680f4423ca2acf89231555787 accessed on 21 February 2025). Please note that the demo dataset is artificially generated and does not correspond to the dataset used in this study.

### 2.2. Variables

This approach used all the available background information after anonymization to predict applicant enrollment. The eight predictor variables, along with their operationalizations, are listed in Table 2. Before the subsequent preprocessing steps, we visualized the proportion of missing values across all predictors and all potential applicants, noting that the incompleteness for both was below 5%. Given that many ML techniques require complete datasets and do not allow for multiple imputations, we used the R package missForest (Stekhoven & Bühlmann, 2012) to impute the missing data. missForest is a single imputation method with an effectiveness comparable to that of multiple imputations. Afterward, continuous predictors retained their original values, whereas categorical predictors were dummy-coded with the first category as the reference. The dependent variable, which represented acceptance of an offer, was coded as 0 if the applicant did not enroll and coded as 1 if the applicant accepted the offer and enrolled. Due to the almost balanced distribution of the class label of the dependent variable, we refrained from using additional sampling strategies that deviated from previous studies (Tate et al., 2020). Prior research has demonstrated that models that address class imbalance do not improve the area under the receiver operating characteristic (ROC) curve (AUC) when compared to models trained without such corrections (van den Goorbergh et al., 2022). More importantly, methods designed to address class imbalance can negatively impact the probability predictions (van den Goorbergh et al., 2022), which are a central focus of our approach.

### 2.3. The First Step: Prediction Model Training

#### 2.3.1. Classification Algorithms

Given the binary nature of our outcome variable, we used four widely accepted ML classification algorithms that were found to be effective in previous research (Nieto et al., 2019).

Logistic regression (LogReg): As it is a foundational algorithm in statistical learning (Etzler et al., 2024), we initially applied linear logistic regression. However, with numerous predictors and the potential for collinearity, the risk of overfitting in LogReg was clear (Shipe et al., 2019).

Elastic Net: To mitigate the risks associated with LogReg, we used the Elastic Net algorithm, a regularized regression technique that combines the benefits of Ridge and LASSO regression (Pargent & Albert-von Der Gönna, 2018). Elastic Net manages multicollinearity through an L1 penalty, akin to Ridge regression, while paralleling LASSO’s propensity to produce a sparse solution through an L2 penalty (Chan et al., 2022).

Classification tree (Ctree): Although beneficial, LogReg and Elastic Net assume a generalized linear relationship between the predictors and outcomes (Chamlal et al., 2024). In situations in which linearity is not evident, Ctree offers an advantage by allowing for nonlinear and interaction effects without requiring strong assumptions about the relationship between the predictors and outcomes (Dumitrescu et al., 2022).

Random Forest (RF): Although Ctree detects nonlinear and interaction effects, its sensitivity to slight variations in the training data limits its stability. To address this limitation, we integrated the RF algorithm, known for its ensemble of bagged and decor-related classification trees, to improve the robustness of the predictions. Previous research underscores RF’s ability to significantly improve the predictive performance, especially when considering nonlinear and interaction effects (Fife & D’Onofrio, 2023).

#### 2.3.2. Hyperparameter Tuning

Hyperparameter tuning is critical to optimizing the performance of ML models (Arnold et al., 2024), especially their discrimination. For LogReg, the hyperparameters do not need to be manually specified. However, other ML algorithms often require fine-tuning of the hyperparameters (Rosenbusch et al., 2021). Grid search is a hyperparameter optimization algorithm. It exhaustively searches for hyperparameters from a specified, explicitly configured grid space (Pargent et al., 2023). We used a grid search in which each potential hyperparameter type was searched for in a semi-random grid of 20 candidate parameters. We combined this with tenfold cross-validation, repeated ten times on the training data. The hyperparameter configuration with the best average performance across the cross-validation folds was selected as the optimal hyperparameter set for each classification algorithm.

For Elastic Net, we adjusted the regularization parameter, often referred to as lambda, and the mixture parameter, which determines the balance between the L1 and L2 penalties. For Ctree, we adjusted the complexity parameter, which dictates the tree splits, the maximum tree depth, and the minimum number of samples present in a node. For RF, we additionally tuned the tree numbers and the number of predictors selected for each split within a single decision tree.

#### 2.3.3. Model Calibration

Our approach emphasizes not only the accuracy of binary classification predictions (discrimination) but also the precision of probabilistic predictions (calibration). Whereas discrimination refers to the ability of a model to separate data into different classes, calibration refers to how closely a model’s predicted probabilities align with the true underlying probabilities of the event (Lindhiem et al., 2020). This focus is essential, as our suggested approach involves predicting the individual enrollment probabilities to estimate the total number of enrolled applicants (*E*) and the probability of this number exceeding the available study places (P(E>SP|A)). Calibration and discrimination are distinct yet complementary aspects of model accuracy. A model might show strong discrimination by correctly ranking individuals based on event probabilities but might fail in calibration, systematically overestimating or underestimating the event probabilities (Jiang et al., 2012). Therefore, appropriate model calibration is essential to ensure reliable and interpretable probability predictions in our approach.

To achieve this, we first identified the best-performing model for each algorithm type by selecting the optimal hyperparameter configuration through the hyperparameter tuning process. Following a similar procedure, we compared three model calibration methods—logistic regression, isotonic regression, and beta calibration—based on the out-of-fold (OOF) predictions generated from tenfold cross-validation, repeated ten times on the training data. The best calibration method within each type of algorithm, representing the best overall calibration performance, was incorporated into the optimal hyperparameter set for the corresponding classification algorithm and applied in subsequent steps.

#### 2.3.4. Performance Comparison

We evaluated four ML classification algorithms using two key metrics to identify the model that achieved the optimal balance between discriminatory power and probability prediction accuracy. During hyperparameter tuning, the AUC was used to compare the algorithm types. Within each algorithm type, the Brier score was used to compare the calibration methods. After the model training, both metrics were calculated with 95% confidence intervals (CIs) on the test dataset to evaluate the generalization performance of the best configuration from each of the four algorithms, incorporating both the optimal hyperparameter tuning and model calibration. This workflow is commonly used in ML studies, particularly those focused on probabilistic predictions (e.g., Tennenhouse et al., 2020; Walsh et al., 2017).

AUC: The AUC is a widely accepted performance measure that evaluates the area under the ROC curve (Orrù et al., 2020). The ROC curve plots the true positive rate (TPR or sensitivity; the proportion of correctly classified enrollees among those who enrolled) against the false positive rate (FPR or specificity; the proportion of applicants incorrectly classified as enrollees among those who declined their offers). The AUC provides a comprehensive performance summary across all potential thresholds without needing to dichotomize continuous values (Swets, 1988). An AUC value of 0.5 indicates a classification performance equivalent to random guessing, while values close to 1 indicate very accurate classification.

Brier score: The Brier score is a widely used metric for assessing the accuracy of probabilistic predictions, with a particular focus on accurate probability predictions (Brier, 1950). It measures the mean squared difference between the predicted probabilities and the actual binary outcomes, offering a direct evaluation of how well the predicted probabilities align with the observed frequencies. Lower Brier scores indicate better calibration, with a perfect score of 0 representing a model in which the predicted probabilities match the true outcomes exactly.

### 2.4. The Second Step: Calculating the Expected Number of Enrolled Applicants

On identifying the best prediction model from these four classification algorithms, we used it to predict the probability of each admitted applicant actually enrolling. Subsequently, we obtained the expected number of enrolled applicants ($\hat{E}$) for any possible number of admitted applicants (*A*). The question of calculating this number can be naturally framed as a Poisson binomial distribution problem. Specifically, each admitted applicant’s enrollment decision is represented by a Bernoulli random variable, $X_{i}$, where $X_{i}=1$ if the *i*-th applicant enrolls and $X_{i}=0$ otherwise. The unique success probability for each applicant is denoted by $p_{i}$, as predicted by the model. Unlike a standard binomial distribution, where all trials are assumed to have the same probability of success, the Poisson binomial distribution allows for varying probabilities across trials, which aligns perfectly with the heterogeneous enrollment probabilities across admitted applicants.

Therefore, $\hat{E}$ corresponds to the expectation of the Poisson binomial distribution, expressed as the sum of these independent but nonidentically distributed Bernoulli random variables:

(4) $$\hat{E}=\sum_{i=1}^{A}p_{i},$$

where *A* represents the total number of admitted applicants. In this context, the number of admitted applicants (*A*) corresponds to the top A applicants in the rank-ordered list of applicants generated during the first phase. In other words, we calculated the expected number of enrolled applicants based on this possible number of admitted applicants.

To this end, we first ordered the admitted students based on the rank-ordered list of applicants, and then, for each possible number of admitted applicants, we predicted the expected number of enrollments in the test dataset. Then, to evaluate the performance of this step, we compared the predicted number with the observed enrollment number based on each possible number of admitted applicants. The mean absolute difference across all possible numbers of admitted applicants was calculated.

While the expected number of enrolled applicants ($\hat{E}$) provides a useful estimate, it does not capture the uncertainty or variability in the enrollment numbers. To address this, we used the probability mass function (PMF) of the Poisson binomial distribution to calculate the probability of obtaining a specific number of enrollments based on the top *A* applicants, which would represent the number of admitted applicants. If the number of available study places is fixed, the number of enrollments equaling the available study places P(E=SP|A) can easily be calculated, thereby supporting more informed decision-making. The equation is

(5) P(E=SP)=∑A∈{1,⋯,A}SP∏i∈Api∏j∉A(1−pj),

where {1,⋯,A}SP represents the collection of all subsets of size SP selected from the total *A* applicants.

Additionally, the persons who need to decide upon the number of admitted applicants need to know how large the probability of more applicants enrolling than study places are available is, as well as its complement. We derived the cumulative distribution function (CDF) from the PMF. Using the CDF, we calculated the probability of enrolling within the capacity as

(6) $$P\left(E\leq SP|A\right)=\sum_{E=0}^{SP}P\left(E\right)$$

and the probability of exceeding capacity as

(7) P(E>SP|A)=1−P(E≤SP|A)

Furthermore, using these two probabilities, we also calculated the 95% CI for *E*. The lower bound of the CI corresponded to the smallest *E* for P(E≤lowerbound|A)≥2.5%, and the upper bound was the largest *E* for P(E≤upperbound|A)≤97.5%. These probabilities and CIs are essential for guiding decisions on the number of admissions, balancing the trade-off between maximizing enrollment and avoiding overcapacity.

To evaluate the performance of using this 95% CI, we used the observed enrollment number to calculate the interval coverage, which measured the proportion of observed enrollment numbers that fell within the 95% CI.

### 2.5. The Third Step: Bootstrapping to Derive Measures for Statistical Uncertainty

Probabilistic predictions in ML are inherently nondeterministic, reflecting the uncertainties associated with the prediction model and the data (Ghahramani, 2015). To account for uncertainties, we extended the Poisson binomial distribution by introducing a mixture Poisson binomial distribution, where the event probabilities ($p_{i}$) were treated as random variables rather than as fixed values. This approach captures both the individual-level variability and the uncertainty in the probability estimates, providing a more robust and realistic characterization of the prediction of the enrollment numbers.

The model’s prediction uncertainty can be quantified through the prediction distribution, which represents the variability in the predicted values for the test dataset based on the training dataset (He & Jiang, 2023). While closed-form solutions exist for simple models such as linear regression, more complex classification algorithms require computational methods (Kompa et al., 2021; Pevec & Kononenko, 2015). Bootstrapping, a model-agnostic approach, is widely used and is based on the premise that the distribution of a given statistic can be approximated according to the empirical distribution of this statistic across resampled subsets (Osband, 2016). In our context, multiple bootstrap samples were generated from the training dataset, and for each sample, a model was trained to produce a single Poisson binomial distribution that reflected the variability in the predicted probabilities. The mixture Poisson binomial distribution, which incorporates both the individual variability and the model’s uncertainty, was then approximated by aggregating the Poisson binomial distributions from all of the bootstrap samples. Specifically, the expectation, PMF, and CDF of the mixture distribution were estimated as the averages of their counterparts from the individual bootstrap samples.

As a result, all of the calculations described in Step 2 explicitly incorporated the uncertainty in the model’s probability estimates through bootstrapping. The results presented in the Section 3, including the estimates of the enrollment outcomes, the probabilities, and the CIs, are derived from the bootstrapped calculations, ensuring that both the model’s uncertainty and sampling uncertainty are fully accounted for in the analysis.

### 2.6. Comparison of the Suggested Approach with the Traditional Approach

To provide insights into the performance of the suggested approach, first, we present the results with a fixed number of admitted applicants determined through selection using the traditional approach. Specifically, in our example, to ensure adequate enrollment in the year 2020, the traditional approach used the enrollment yield from the average yield from 2017 to 2019 (EY=45.68%). To illustrate the comparison, we considered a hypothetical scenario in which the study program had only 50 available study places in 2020. A small number of SP was necessary in this example because we needed empirical enrollment data for all students who might have enrolled to compare the suggested approach with the traditional approach. Because we only had empirical enrollment data for 85 students, we chose SP=50 here. The calculation suggested that at least A=50/45.68×100≈110 applicants needed to be admitted. For this value of *A*, we calculated the probability of this number exceeding the number of study places using the suggested ML-based approach.

Next, the ML-based prediction model was employed to estimate *A* for two scenarios: a low- (P(E>SP|A)=5%) and a high-risk preference (P(E>SP|A)=20%) regarding the probability of enrolling too many students.

Then, for the target number of occupied study places mentioned before (SP=50), we directly compared the enrollment yield (EY) achieved by the traditional and the ML-based prediction models with a risk preference of P(E>SP|A)=10%, which we deemed to be a realistic value in many applications.

Subsequently, we compared the ability of the traditional and ML-based approaches to capture the local fluctuations in the enrollment numbers. Instead of focusing solely on the overall prediction error, we evaluated how well each approach captured the local variations by computing the mean rolling correlation between the predicted and observed *E* across all possible numbers of admitted applicants. This analysis enabled us to assess which approach more accurately reflected the year-specific application trends (e.g., more/fewer students who had obtained their bachelor’s degree from a university), providing a more reliable basis for practical decision-making.

### 2.7. Visualization

Visualizing probabilities is helpful for wise decision-making (Joslyn & Savelli, 2021). Therefore, we developed a Shiny app that could be employed on a local device and provide admission officers with a graphical tool to connect the above-mentioned expected number $\hat{E}$ and the related probability P(E>SP∣A).

## 3. Results

### 3.1. The First Step: Prediction Model Training

The results presented are based on the best-performing model configurations and calibration methods identified during the model training. All of the metrics reported for the test dataset incorporate these optimizations. The results obtained for the four classification algorithms when used to predict the applicant enrollment in 2020 are shown in Table 3.

Each algorithm has a point estimate and a 95% CI for the Brier score. Given that the 95% CIs for the Brier scores did not include the value of 0.25 (the expected score for random guessing), all of the classification algorithms demonstrated significantly better probabilistic accuracy than random guessing. The algorithms showed accuracies in the range of 0.15 to 0.17, with the differences between the algorithms being nonsignificant (*p* > 0.05) using the same procedure for the AUC.

**Figure 1:**
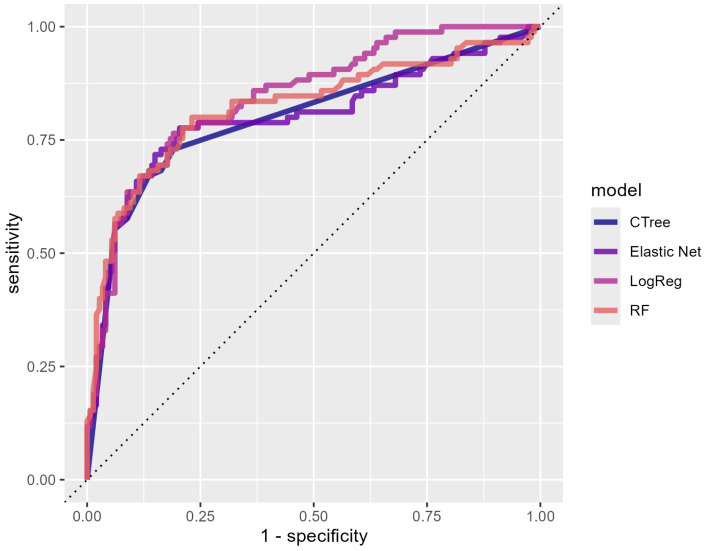
AUC plot for logistic Regression (LogReg), Elastic Net, Classification Tree (Ctree), and Random Forest (RF) used to predict applicant enrollment in 2020.

A comparable pattern was observed for the AUC measures. Given that the 95% confidence intervals did not cover the value of 0.5, all of the classification algorithms significantly outperformed random guessing. Additionally, for none of the algorithms did the AUC point estimate fall outside of the 95% CI of the AUC of the other algorithms. Therefore, the differences in the AUCs were not significant (*p* > 0.05). We present the ROC curves to visualize our results (Figure 1), where the *x*-axis represents sensitivity and the *y*-axis represents 1-specificity. Different colors represent different algorithms. The closeness of these curves suggests that no single algorithm clearly outperformed the others. A slight difference can be observed at higher 1-specificity values, where logistic regression exhibits a marginally better sensitivity compared to that of the other algorithms, resulting in an ROC curve that appears to cover a slightly larger area. However, this difference was not statistically significant (*p* > 0.05), indicating that the overall performance remained comparable across the models.

Although all of the classification algorithms showed comparable classification abilities for the data used, we had to choose one for the next step. We chose the Random Forest (RF) algorithm. This choice was made for two reasons: First, RF requires fewer assumptions than its counterparts (Fife & D’Onofrio, 2023). Second, RF lacks a closed-form solution for computing the prediction distribution, so it can be used to illustrate the full suggested approach, including the bootstrap step.

Using this model, the mean probability of an applicant enrolling after being offered a study place in 2020 was 0.43 (SD = 0.30), with a range of 0.16 to 0.94. Thus, the individual probabilities varied substantially, underlining the necessity of the suggested approach.

### 3.2. The Second and Third Steps: The Expected Number of Enrolled Applicants and Probability Estimation After Accounting for Uncertainty

In the second step, we used the RF algorithm to predict the enrollment of the applicants, sorted according to the rank-ordered list of applicants generated during the first phase. Next, we calculated the expected number of enrollments ($\hat{E}$) among the 232 individuals for whom enrollment data were available in the dataset. Notably, all of the results presented here were obtained using bootstrapping to account for the uncertainty in the model’s probabilistic predictions. The mean difference between the predicted and observed enrollment numbers across the 232 levels of *A* was 9.05 (SD=3.89; range: −0.10 to 14.49). For most values of *A*, the predicted numbers of enrolled applicants nominally showed slightly higher values than the observed numbers. These results indicate that although the deviations in the point estimates of *E* were not that large, there was evidence of systematic bias. This suggests a consistent misalignment between the predicted and observed enrollment numbers across a wide range of possible decisions regarding *A*.

**Figure 2:**
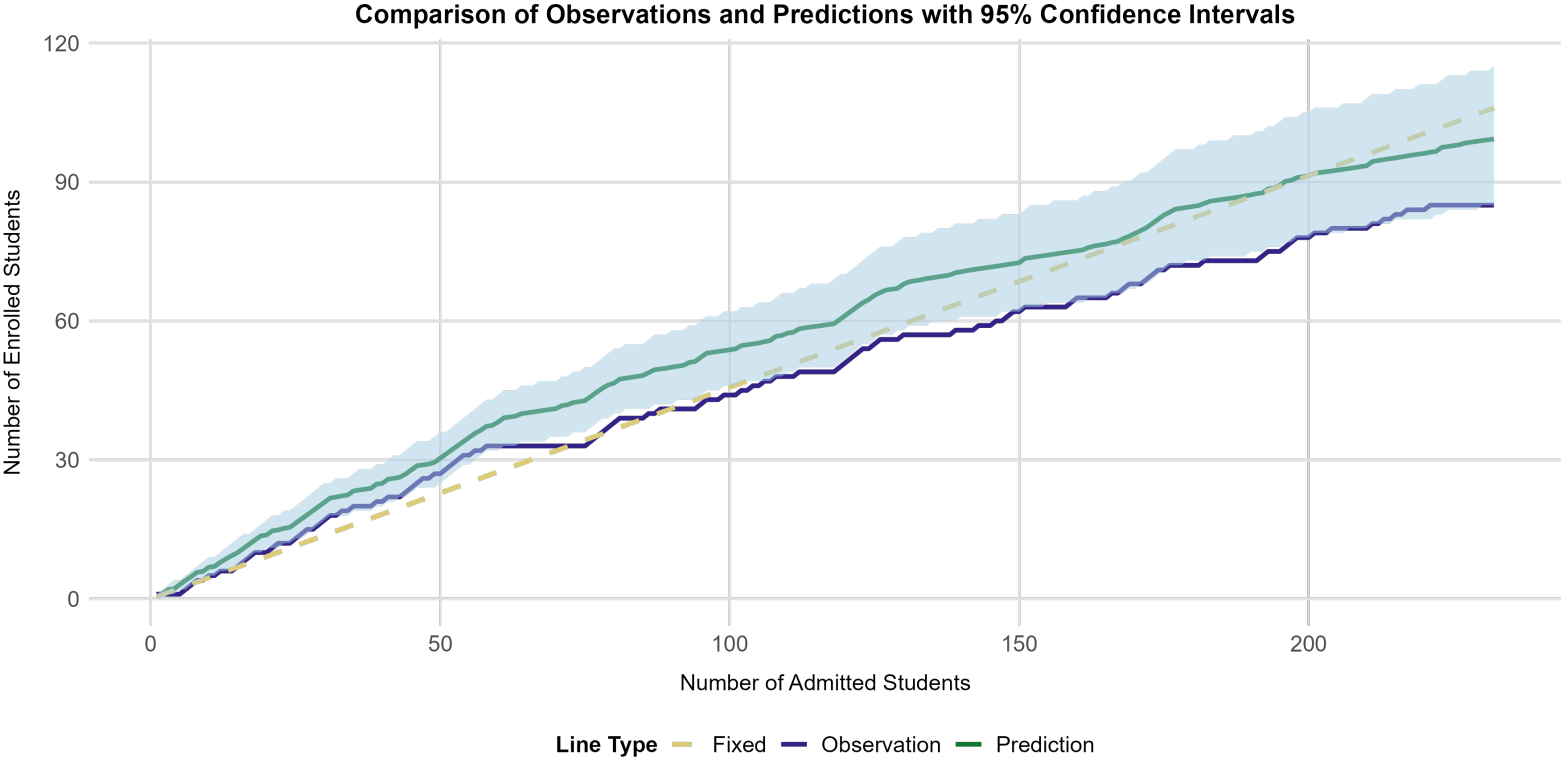
The relationship between the numbers of admitted applicants and the predicted enrollment numbers. The 95% confidence interval, calculated from the 95% percentiles of the distribution, is shown, capturing the variability in the predictions. For comparison, the traditional approach with a fixed enrollment yield (EY) based on the 2017–2019 average is represented by a dashed line as a reference.

Subsequently, we used the 95% CI to show the variability in the enrollment numbers. Both the observed and predicted numbers of enrolled applicants conditional upon the number of admitted applicants are shown in Figure 2. The blue-shaded area represents the 95% CI of $\hat{E}$. The interval becomes wider with increasing *A* values. This is expected because the prediction becomes more difficult (resulting in increasing statistical uncertainty) when more applicants with an individual probability of enrolling are admitted. More than half (52.15%) of the observed *E* values fell into this CI across the 232 levels of *A*, indicating that the differences between $\hat{E}$ and the observed enrollment numbers were not significant in at least half of the cases. This result suggests that our model has some predictive power, although caution is warranted.

### 3.3. Comparison of the Suggested Approach with the Traditional Approach

For a fixed number of admitted applicants (A=110) determined through the traditional approach, the ML-based approach yielded a probability of exceeding the target number of enrolled applicants P(E>SP∣A)=94.13%. This result highlights that the traditional approach leads to a very high risk of overenrollment.

Using the ML-based approach, we also estimated the optimal number of admitted applicants (*A*) required to achieve specific probabilities of overenrollment under two risk scenarios: For the low-risk preference P(E>SP|A)=5%, the ML-based approach suggested admitting at least 78 applicants. For the high-risk preference P(E>SP|A)=20%, the ML-based approach suggested admitting at least 82 applicants. These values are smaller than the fixed *A* obtained using the traditional approach.

Further, we compared the EY of the traditional approach with that of the ML-based approach for a moderate-risk preference of P(E>SP∣A)=10%. Following the traditional approach, A=50/45.68×100≈110 applicants ought to have been admitted, and of these, only 48 ultimately enrolled, resulting in an EY of 48/110≈43.6%. By contrast, the ML-based approach suggested admitting 79 students for the moderate-risk preference. Of the top 79 admitted applicants, 37 ultimately enrolled, yielding a higher enrollment yield of 37/79≈46.8%. This improvement underscores the effectiveness of the ML-based approach in optimizing admission decisions and improving enrollment outcomes.

Afterward, we assessed the ability of the traditional and ML-based approaches to capture the local fluctuations in *E* by computing the rolling correlation between the predicted and observed *E* across all possible numbers of *A*. Using a window of size of 10, the ML-based approach demonstrated stronger alignment with the local change in the observed *E*, as indicated by a higher mean rolling correlation (*M* = 0.96), compared to that of the traditional approach (*M* = 0.94). These results suggest that the ML-based approach more accurately reflects local variations in enrollment, making it a more reliable tool for practical applications in admission forecasting.

### 3.4. Visualization

A Shiny app was created using R (Version 4.4.2) to give the people responsible for student admissions the possibility to apply the suggested approach. Usage of the app does not require any methodological or statistical training. The purpose of this app is to inform decision-makers about the number of applicants to admit. It accomplishes this by allowing them to enter the number of study places and to adjust the number of applicants admitted. The resulting predicted probabilities are that (a) fewer applicants will enroll than there are study places (P(E<SP∣A)), (b) exactly as many applicants will enroll as there are study places ( (P(E=SP∣A)), and (c) more applicants will enroll than there are study places (P(E>SP∣A)) are calculated instantaneously and represented numerically and as a pie chart.

**Figure 3:**
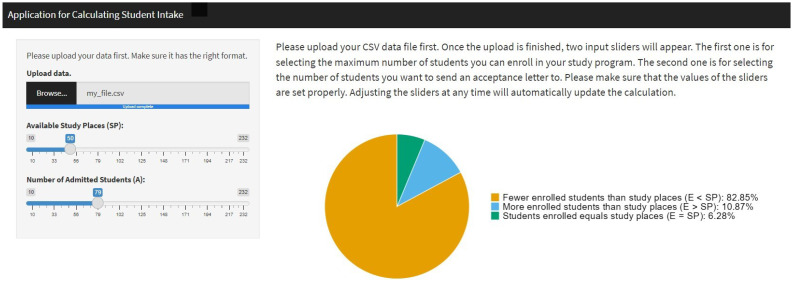
Screenshot of Shiny app for visualization.

First, the user is asked to upload a comma-separated values (*.csv) file. This data file should include the information gained from the bootstrapping algorithm described above. Thus, a methodologically trained person has to train a prediction model and perform the bootstrapping beforehand. As soon as the data file is uploaded, the user is presented with two input sliders on the left side of the screen: One of these sliders asks for the number of study places (SP) available; the other slider asks for the number of applicants to be admitted (A). The user interface displays the resulting predicted probabilities numerically and the pie chart (see Figure 3) on the right side of the screen. The information is updated automatically whenever the input provided by the user changes.

The OSF repository provides a more detailed description of the format that the *.csv file should have and the code for running the app locally.

## 4. Discussion

This paper introduces and illustrates a novel ML-based approach to informing student admission decisions. The results show that the suggested approach improves upon the enrollment outcomes from the traditional approach that does not rely on a prediction model.

In Step 1 of the suggested approach, several ML-based models are trained, making use of the applicants’ background information to predict the individual enrollment probabilities. We employed and compared multiple algorithms and evaluated the predictive model performance in this step. The performance of the four algorithms was similar for the dataset we used. All of the models resulted in substantial variability in the individual enrollment probabilities; this indicates that using statistical modeling to predict whether an individual will enroll if they are offered a study place is a promising approach to improving the admission process.

In the second and third steps, our approach can predict the expected number of enrolled applicants $\hat{E}$ as a function of the number of admitted applicants *A* by accumulating all of the individual predicted enrollment probabilities. By incorporating the statistical uncertainty of these individual predictions, this approach provides the probabilities of too many, just enough, or too few applicants being enrolled. This capability offers valuable support for admission managers, enabling dynamic and data-driven adjustments to the number of admitted applicants (*A*) based on institutional goals and risk preferences. Such information is not available in the traditional approach.

The evaluation of the test dataset showed good predictive performance, with the predicted enrollment numbers aligning with the observed values across all possible numbers of admitted applicants. However, the observed deviations, although averaging fewer than 10, indicate systematic bias that cannot be overlooked. Notably, the predicted enrollment numbers were consistently slightly higher than the observed values, particularly for larger values of *A*. This bias is likely due to differences between the training data (2017–2019) and the test dataset (2020). The 2020 enrollment yield was indeed lower than that in previous years, which may explain the tendency of our model to overestimate the enrollment numbers. One possible reason for the relatively large jump in the EY could be that 2020 was the first year of the COVID-19 pandemic. It is likely that applicants generally felt considerably insecure during this period. Therefore, they may have found it more difficult to decide to enroll and actually start their studies, which often would have involved finding housing and relocating, for example. Under the circumstances without such an extreme situation as a pandemic, we expect a more stable EY and even better performance of the ML-based approach. Nevertheless, this result also points to the fact that the suggested ML-based approach produces the best predictions when the EY is relatively stable.

It has to be noted, though, that systematic overestimation was not unique to our approach. Even when a traditional approach with the enrollment yield based on historical data was used, overestimation resulted. However, the results of the rolling correlation demonstrated that our method more accurately captured the local variations in the enrollment numbers, producing predicted numbers much closer to the observed values. This suggests that despite the systematic bias, our approach is better suited to capturing year-specific fluctuations in the proportions of groups of applicants with differing enrollment yields. Future work should explore methods to reduce this bias further, particularly when large differences exist between the EYs of the training and test datasets.

Moreover, the results also showed that the traditional approach exhibited an obvious risk of overenrollment (P(E>SP∣A)=94.13%), whereas the ML-based approach made it possible to quantify the risk involved in the admission procedure (e.g., the probability of more students enrolling than predicted or exceeding the available study places). Under a moderate-risk scenario (10% risk of too many students being enrolled), the ML-based approach demonstrated an advantage by achieving a higher enrollment yield. Obviously, the difference between the enrollment yield using the traditional and ML-based approaches depends upon the willingness of the people making the admission decisions to take risks. This approach makes it possible to work with probabilities, mitigating the risk of enrolling too few or too many students. This ensures that over- or underfilling degree programs are no longer contingent on the risk preferences of those making the admission decisions but rather on a structured, data-driven process.

### 4.1. Sample Size Requirements

The effectiveness of the suggested approach depends on the availability of a sufficiently large dataset (Vabalas et al., 2019). Small sample sizes in datasets may reduce a model’s predictive performance and increase the risk of overfitting, where the model learns patterns specific to the training data but fails to generalize well to new data. In our application, we had a dataset of n=996, which was relatively small for complex ML models (Vabalas et al., 2019). Nevertheless, even with this dataset, we obtained relatively accurate and precise estimates of the number of enrolled applicants conditional upon admission. While the model exhibits some systematic bias, particularly in slightly overestimating the enrollments, its ability to capture year-specific local enrollment fluctuations and quantify risk preferences makes it a valuable tool for admission forecasting.

Although our results demonstrate the feasibility of applying ML in student admission with a relatively small dataset, the accuracy and generalizability to future years of such an approach could be improved with larger sample sizes. A larger dataset would reduce the variance, enhance the model’s stability, and lower the risk of overfitting, leading to more precise and robust predictions (Pate et al., 2020). In addition, if more years of data were available, the ML-based approach could capture generalizable patterns in the enrollment processes better, reducing the influence of any single year’s unique circumstances. Future research should explore the impacts of including more data on predictive accuracy and investigate strategies for incorporating additional data sources to enhance the performance of our approach.

### 4.2. Model Comparison in the First Step

Our results indicated that the four ML algorithms showed comparable performance, as there was limited variability in their outcomes across different algorithms. Similar conclusions have been reported in previous studies, which found minimal differences in different algorithms’ performance when applied to datasets in psychology (Etzler et al., 2024; Jankowsky et al., 2024). This raises the question of whether the model comparison in Step 1 is necessary or whether this part could be streamlined by directly adopting a single algorithm, such as RF.

Although RF can handle nonlinear relationships and high-dimensional interactions with relatively relaxed assumptions, we argue that this step of the ML-based approach cannot simply be omitted by deciding to adopt a single algorithm. Although the four ML algorithms tested in this study produced similar results, this consistency may have been specific to our dataset and context. It cannot be assumed that such similarity in performance would hold across all datasets with different structures or complexities. This idea aligns with the “No Free Lunch Theorem” (Tate et al., 2020), which posits that no single algorithm universally outperforms others across all problems. Therefore, retaining Step 1 for model comparison is essential to ensure the robustness and adaptability of our suggested approach, particularly in settings with varying data characteristics in different institutions.

### 4.3. Practical Contributions

Our findings highlight that the suggested ML-based approach effectively addresses two challenges in student admission procedures (Shao et al., 2022; Walczak & Sincich, 1999): overly cautious decisions (resulting in unnecessarily low probabilities of meeting the enrollment targets) and hazardous decisions (leading to a high probability of overenrollment). The model enables a balanced strategy that aligns with institutional risk preferences while also adapting to the specific circumstances of different institutions and study programs by dynamically adjusting the number of admitted applicants based on the predicted probabilities of enrollment.

In contrast, the traditional approach, which relies on a value for *A* determined by the previous year’s enrollment yield, often fails to optimize these trade-offs. For example, in our study with 50 predefined study places, the probability of overenrollment using *A* in the traditional approach was extremely high. Our results suggest that slightly reducing *A* would have maintained a controlled probability of overenrollment, remaining below a maximum risk of 10%.

### 4.4. Limitations

While our study demonstrates the successful application of a machine-learning-based approach to student admissions, certain limitations should be acknowledged. First, it has to be noted that our analysis is based on a dataset from one study program and cannot be generalized to other study programs at other universities. However, the underlying principles of our approach, using machine learning and probabilistic prediction to aid student admission decisions, are applicable across study programs and universities as long as the applicants can be rank-ordered according to one or several selection criteria and a fixed number of SP is used. It would be interesting to see the performance of this approach when applied in other institutional settings.

Second, our approach needs admission officers to know the total number of applicants before making decisions. Therefore, it is not useful for institutions with rolling admissions, where applications are admitted continuously rather than on a specific date.

Third, our ML-based approach might raise ethical considerations regarding fairness and potential biases in data-driven decision-making. While ML models enable a more balanced admission strategy, they can also reflect potential biases in historical data. However, our approach does not exacerbate these biases compared to the traditional approach, which relies on the previous year’s enrollment yield, because it does not directly modify individual admission decisions or selection criteria but rather optimizes only *A* based on the predicted probabilities. This ensures that institutional policies, rather than our ML-based approach itself, remain the primary determinant of selection fairness and diversity. Furthermore, while our study focuses on optimizing the enrollment predictions, future research should examine how this ML-based approach affects student diversity, particularly in terms of socioeconomic and demographic composition.

### 4.5. Conclusions

In this study, we introduce a novel ML-based approach to optimizing university admission decisions, addressing the limitations of the traditional approach. The latter explicitly or implicitly relies on the enrollment yields from the previous year, which may not accurately reflect year-to-year variations. By leveraging individual enrollment probabilities and quantifying the statistical uncertainty, this approach, with three steps, enables dynamic, data-driven adjustments to the number of admitted students. Our results show that this ML-based approach enhances the efficiency and flexibility of the admission process while mitigating the risk of both underenrollment and overenrollment. Compared to the traditional approach, this approach adapts to variability in the individual enrollment probabilities, improving decision-making in university admission decisions and providing a robust framework for managing enrollment scenarios in higher education.

## Figures and Tables

**Table 1:** Key metrics for admission and enrollment by year.

Year	Total Number of Applicants (*T*)	Study Places (SP)	Admitted Applicants (*A*)	Admission Rate (AR)	Enrolled Applicants (*E*)	Enrollment Yield (EY)
2017	1546	120	274	17.7%	117	42.7%
2018	1564	120	270	17.3%	114	42.2%
2019	1475	120	220	14.9%	118	53.6%
2020	1726	120	232	13.4%	85	36.6%

*Note.* Number of admitted applicants (*A*), admission rate (AR), enrolled applicants (*E*), and enrollment yield (EY) refer to the first admission round.

**Table 2:** Variable operationalizations and types of measurement.

Variable	Operationalization	Scale of Measurement
Age	Applicants’ age on the first day of the academic year they applied for (e.g., in years).	Ratio
Gender	Identification of the applicants’ gender (e.g., male, female, nonbinary, etc.).	Nominal
Bachelor Location	Classification of the geographical location of applicants’ bachelor’s degree (domestic or international).	Nominal
Undergraduate Institution Status	The institution where the applicant completed their undergraduate degree (same university or different institution).	Nominal
Biographical Origin	The applicants’ country or region of origin (Germany or abroad).	Nominal
Bachelor Major	The major or specialization of the completed bachelor’s degree (general psychology or applied psychology).	Nominal
Commuting Distance	The straight-line distance from the applicants’ home to the university. For EU non-German addresses, the distance from the applicant’s city to the university is calculated. For overseas addresses, the distance is calculated from the nearest point of entry in Germany.	Ratio
Total Number of Applicants (*T*)	Total number of applicants who applied for the academic year.	Ratio

**Table 3:** Prediction model comparison table.

Algorithms	Brier Score (Point Estimate [95% CI])	AUC (Point Estimate [95% CI])
LogReg	0.16 [0.13, 0.19]	0.84 [0.79, 0.90]
Elastic Net	0.16 [0.13, 0.19]	0.80 [0.73, 0.87]
Ctree	0.17 [0.14, 0.20]	0.80 [0.74, 0.86]
RF	0.15 [0.12, 0.19]	0.82 [0.76, 0.88]

*Note.* Point estimates with 95% confidence intervals (CIs) are reported. LogReg = logistic regression; Ctree = classification tree; RF = Random Forest; AUC = area under the receiver operating characteristic curve.

## Data Availability

The R code and a demo dataset for implementing the proposed approach are available in the OSF repository (https://osf.io/qsvcj/?view_only=9b27e0c680f4423ca2acf89231555787, accessed on 21 February 2025). Please note that the demo dataset is simulated and intended for illustrative purposes only and therefore does not correspond to the dataset used in this study. The real dataset used cannot be shared due to data protection regulations.

## Abbreviations

The following abbreviations are used in this manuscript:

US	United States
GPA	grade point average
GDPR	General Data Protection Regulation
ML	machine learning
AUC	area under the curve
ROC	receiver operating characteristic
LogReg	logistic regression
Ctree	classification tree
RF	Random Forest
OOF	out-of-fold
CIs	confidence intervals
TPR	true positive rate
FPR	false positive rate
PMF	probability mass function
CDF	cumulative distribution function
CSV	comma-separated values
T	total number of applicants
SP	study places
A	admitted applicants
AR	admission rate
E	enrolled applicants
EY	enrollment yield

## References

[B1-behavsci-15-00330] Arnold C., Biedebach L., Küpfer A., Neunhoeffer M. (2024). The role of hyperparameters in machine learning models and how to tune them. Political Science Research and Methods.

[B2-behavsci-15-00330] Brier G. W. (1950). Verification of forecasts expressed in terms of probability. Monthly Weather Review.

[B3-behavsci-15-00330] Briihl D. S., Wasieleski D. T. (2004). A survey of master’s-level psychology programs: Admissions criteria and program policies. Teaching of Psychology.

[B4-behavsci-15-00330] Bruggink T. H., Gambhir V. (1996). Statistical models for college admission and enrollment: A case study for a selective liberal arts college. Research in Higher Education.

[B5-behavsci-15-00330] Chamlal H., Benzmane A., Ouaderhman T. (2024). Elastic net-based high dimensional data selection for regression. Expert Systems with Applications.

[B6-behavsci-15-00330] Chan J. Y.-L., Leow S. M. H., Bea K. T., Cheng W. K., Phoong S. W., Hong Z.-W., Chen Y.-L. (2022). Mitigating the multicollinearity problem and its machine learning approach: A review. Mathematics.

[B7-behavsci-15-00330] Dumitrescu E., Hué S., Hurlin C., Tokpavi S. (2022). Machine learning for credit scoring: Improving logistic regression with non-linear decision-tree effects. European Journal of Operational Research.

[B8-behavsci-15-00330] Etzler S., Schönbrodt F. D., Pargent F., Eher R., Rettenberger M. (2024). Machine learning and risk assessment: Random forest does not outperform logistic regression in the prediction of sexual recidivism. Assessment.

[B9-behavsci-15-00330] European Parliament and Council of the European Union (2016). Regulation (EU) 2016/679 of the european parliament and of the council of 27 April 2016 on the protection of natural persons with regard to the processing of personal data and on the free movement of such data, and repealing directive 95/46/EC (general data protection regulation). Official Journal of the European Union L.

[B10-behavsci-15-00330] Fife D. A., D’Onofrio J. (2023). Common, uncommon, and novel applications of random forest in psychological research. Behavior Research Methods.

[B11-behavsci-15-00330] Fu Y. C., Fernandez F., Kao J. H., Tseng K. H. (2022). Does geodemographic segmentation influence higher education opportunity? A spatial investigation of enrollment at one taiwanese university. Higher Education.

[B12-behavsci-15-00330] Ghahramani Z. (2015). Probabilistic machine learning and artificial intelligence. Nature.

[B13-behavsci-15-00330] Goenner C. F., Pauls K. (2006). A predictive model of inquiry to enrollment. Research in Higher Education.

[B14-behavsci-15-00330] He W., Jiang Z. (2023). Uncertainty quantification of deep learning for spatiotemporal data: Challenges and opportunities. arXiv.

[B15-behavsci-15-00330] Jankowsky K., Steger D., Schroeders U. (2024). Predicting lifetime suicide attempts in a community sample of adolescents using machine learning algorithms. Assessment.

[B16-behavsci-15-00330] Jiang X., Osl M., Kim J., Ohno-Machado L. (2012). Calibrating predictive model estimates to support personalized medicine. Journal of the American Medical Informatics Association.

[B17-behavsci-15-00330] Joslyn S., Savelli S. (2021). Visualizing uncertainty for non-expert end users: The challenge of the deterministic construal error. Frontiers in Computer Science.

[B18-behavsci-15-00330] Kompa B., Snoek J., Beam A. L. (2021). Second opinion needed: Communicating uncertainty in medical machine learning. npj Digital Medicine.

[B19-behavsci-15-00330] Li K. C., Wong B. T.-M., Chan H. T., Li C., Cheung S. K. S., Wang F. L., Lu A., Kwok L. F. (2023). Predictive analytics for university student admission: A literature review. Blended learning: Lessons learned and ways forward.

[B20-behavsci-15-00330] Lindhiem O., Petersen I. T., Mentch L. K., Youngstrom E. A. (2020). The importance of calibration in clinical psychology. Assessment.

[B21-behavsci-15-00330] Lotfi V., Maki B. (2018). A predictive model for graduate application to enrollment. Open Access Library Journal.

[B22-behavsci-15-00330] National Center for Education Statistics (n.d.). Back to school statistics.

[B23-behavsci-15-00330] Nieto Y., Gacía-Díaz V., Montenegro C., González C. C., Crespo R. G. (2019). Usage of machine learning for strategic decision making at higher educational institutions. IEEE Access.

[B24-behavsci-15-00330] Nietzel M. T. (2022). Ivy league colleges reveal acceptance numbers for class of 2026.

[B25-behavsci-15-00330] Orrù G., Monaro M., Conversano C., Gemignani A., Sartori G. (2020). Machine learning in psychometrics and psychological research. Frontiers in Psychology.

[B26-behavsci-15-00330] Osband I. (2016). Risk versus uncertainty in deep learning: Bayes, bootstrap and the dangers of dropout. NIPS workshop on bayesian deep learning.

[B27-behavsci-15-00330] Pargent F., Albert-von Der Gönna J. (2018). Predictive modeling with psychological panel data. Zeitschrift für Psychologie.

[B28-behavsci-15-00330] Pargent F., Schoedel R., Stachl C. (2023). Best practices in supervised machine learning: A tutorial for psychologists. Advances in Methods and Practices in Psychological Science.

[B29-behavsci-15-00330] Pate A., Emsley R., Sperrin M., Martin G. P., van Staa T. (2020). Impact of sample size on the stability of risk scores from clinical prediction models: A case study in cardiovascular disease. Diagnostic and Prognostic Research.

[B30-behavsci-15-00330] Pevec D., Kononenko I. (2015). Prediction intervals in supervised learning for model evaluation and discrimination. Applied Intelligence.

[B31-behavsci-15-00330] Rosenbusch H., Soldner F., Evans A. M., Zeelenberg M. (2021). Supervised machine learning methods in psychology: A practical introduction with annotated R code. Social and Personality Psychology Compass.

[B32-behavsci-15-00330] Shao L., Levine R. A., Hyman S., Stronach J., Fan J. (2022). A combinatorial optimization framework for scoring students in university admissions. Evaluation Review.

[B33-behavsci-15-00330] Shipe M. E., Deppen S. A., Farjah F., Grogan E. L. (2019). Developing prediction models for clinical use using logistic regression: An overview. Journal of Thoracic Disease.

[B34-behavsci-15-00330] Shrestha R. M., Orgun M. A., Busch P. (2016). Offer acceptance prediction of academic placement. Neural Computing and Applications.

[B35-behavsci-15-00330] Smith A. B., Street M. A., Olivarez A. (2002). Early, regular, and late registration and community college student success: A case study. Community College Journal of Research and Practice.

[B36-behavsci-15-00330] Stekhoven D. J., Bühlmann P. (2012). MissForest—non-parametric missing value imputation for mixed-type data. Bioinformatics.

[B37-behavsci-15-00330] Swets J. A. (1988). Measuring the accuracy of diagnostic systems. Science.

[B38-behavsci-15-00330] Tate A. E., McCabe R. C., Larsson H., Lundström S., Lichtenstein P., Kuja-Halkola R. (2020). Predicting mental health problems in adolescence using machine learning techniques. PLoS ONE.

[B39-behavsci-15-00330] Tennenhouse L. G., Marrie R. A., Bernstein C. N., Lix L. M., Others (2020). Machine-learning models for depression and anxiety in individuals with immune-mediated inflammatory disease. Journal of Psychosomatic Research.

[B40-behavsci-15-00330] Unangst L. (2019). Refugees in the german higher education system: Implications and recommendations for policy change. Policy Reviews in Higher Education.

[B41-behavsci-15-00330] Vabalas A., Gowen E., Poliakoff E., Casson A. J. (2019). Machine learning algorithm validation with a limited sample size. PLoS ONE.

[B42-behavsci-15-00330] van den Goorbergh R., van Smeden M., Timmerman D., Van Calster B. (2022). The harm of class imbalance corrections for risk prediction models: Illustration and simulation using logistic regression. Journal of the American Medical Informatics Association.

[B43-behavsci-15-00330] Walczak S., Sincich T. (1999). A comparative analysis of regression and neural networks for university admissions. Information Sciences.

[B44-behavsci-15-00330] Walsh C. G., Ribeiro J. D., Franklin J. C. (2017). Predicting risk of suicide attempts over time through machine learning. Clinical Psychological Science.

